# Kisspeptin Signaling Is Required for the Luteinizing Hormone Response in Anestrous Ewes following the Introduction of Males

**DOI:** 10.1371/journal.pone.0057972

**Published:** 2013-02-28

**Authors:** Julie-Ann P. De Bond, Qun Li, Robert P. Millar, Iain J. Clarke, Jeremy T. Smith

**Affiliations:** 1 Department of Physiology, Monash University, Melbourne, Victoria, Australia; 2 Mammal Research Institute, University of Pretoria, Pretoria, Gauteng, South Africa; 3 UCT/MRC Receptor Biology Unit, University of Cape Town, Cape Town, Western Cape, South Africa; 4 Centre for Integrative Physiology, University of Edinburgh, Edinburgh, Scotland; University of Rouen, France

## Abstract

The introduction of a novel male stimulates the hypothalamic-pituitary-gonadal axis of female sheep during seasonal anestrus, leading to the resumption of follicle maturation and ovulation. How this pheromone cue activates pulsatile secretion of gonadotropin releasing hormone (GnRH)/luteinizing hormone (LH) is unknown. We hypothesised that pheromones activate kisspeptin neurons, the product of which is critical for the stimulation of GnRH neurons and fertility. During the non-breeding season, female sheep were exposed to novel males and blood samples collected for analysis of plasma LH profiles. Females without exposure to males served as controls. In addition, one hour before male exposure, a kisspeptin antagonist (P-271) or vehicle was infused into the lateral ventricle and continued for the entire period of male exposure. Introduction of a male led to elevated mean LH levels, due to increased LH pulse amplitude and pulse frequency in females, when compared to females not exposed to a male. Infusion of P-271 abolished this effect of male exposure. Brains were collected after the male effect stimulus and we observed an increase in the percentage of kisspeptin neurons co-expressing Fos, by immunohistochemistry. In addition, the per-cell expression of *Kiss1* mRNA was increased in the rostral and mid (but not the caudal) arcuate nucleus (ARC) after male exposure in both aCSF and P-271 treated ewes, but the per-cell content of *neurokinin B* mRNA was decreased. There was also a generalized increase in Fos positive cells in the rostral and mid ARC as well as the ventromedial hypothalamus of females exposed to males. We conclude that introduction of male sheep to seasonally anestrous female sheep activates kisspeptin neurons and other cells in the hypothalamus, leading to increased GnRH/LH secretion.

## Introduction

Reproduction is driven by the pulsatile secretion of gonadotropin-releasing hormone (GnRH) from the hypothalamus. Regulation of the hypothalamo-pituitary gonadal (HPG) axis involves the integration of a series of central neuronal inputs that mediate environmental influences as well as sex steroid feedback and other endogenous factors (metabolic signals, stress hormones etc)[1], [2]. Among the exteroceptive factors are sociosexual stimuli, which include olfactory signals, or pheromones. Pheromones are chemical signals produced by males and females, detected by the olfactory system. In the context of reproductive function, such signals may be relayed to the GnRH cells. The effect of pheromones to stimulate reproduction has been well described in sheep, where introduction of males to previously isolated females leads to an increase in the pulsatile secretion of LH in the latter [3]. This ‘male effect’ (which may not be restricted to pheromones) is able to override the seasonal quiescence in reproductive function in this species, causing ovulation in anestrous females in the non-breeding season.

The precise neuroendocrine pathway in sheep linking pheromones and the olfactory system to GnRH secretion is yet to be accurately determined [4]. Within the brain, assessment of neural activation (via the protein product of immediate early gene *FOS*) has shed light on the possible pathways from olfactory centers converging to GnRH neurons. Pheromonal signals are processed as olfactory stimuli in the brain and processing originates at either the main or accessory olfactory bulbs [5], resulting in neural activation of key structures within both systems then extending to the hypothalamus [6] and ultimately GnRH neurons in the medial preoptic area (mPOA) [7]. Importantly, the precise neuroanatomical pathway and/or neuronal population responsible for transmitting pheromonal stimuli to the GnRH neurons is not known. A recent study in goats demonstrated that male pheromone presentation to females increased the multiple-unit activity [8] within the arcuate nucleus (ARC) thought to represent a GnRH pulse modulator [9]. Moreover, the MUA recordings were noted to be in close proximity to kisspeptin neurons [10], [11] and pheromones have been shown to activate kisspeptin neurons in mice [12]. The product of the *Kiss1* gene, kisspeptin, is one neuropeptide that may provide a link between the olfactory system and GnRH neurons.

Kisspeptin signaling is essential for GnRH secretion and reproduction [13], [14]. In humans and mice, loss of function mutations in the kisspeptin receptor (Kiss1r) result in failure to progress through puberty and resultant infertility [14]. Kisspeptin stimulates LH secretion in a GnRH dependant manner [15] by increasing GnRH secretion into the hypophysial portal blood [16]. In sheep, kisspeptin neurons (those expressing *Kiss1* mRNA) are located in the dorso-lateral preoptic area (POA) and the ARC [17], [18], [19], [20]. In the ARC, kisspeptin neurons express estrogen and progesterone receptors [18], [19] and are directly regulated by these steroids in a manner consistent with both positive and negative feedback regulation of pulsatile GnRH secretion [19], [20], [21], [22], [23]. Alternatively, kisspeptin neurons in the dorso-lateral POA appear to be involved in the positive feedback signal to induced the preovulatory LH surge [21].

Given the role of kisspeptin neurons in the negative feedback effects of sex steroids on GnRH secretion and the role of negative feedback in the seasonal suppression of reproduction [24], it is not surprising that the kisspeptin is proposed to play a key role in the seasonal regulation of reproduction in sheep. *Kiss1* expression and peptide production is markedly up-regulated in the ARC at the onset of the breeding season [19], [20], [25]. In addition, the number of kisspeptin fibers in close apposition to GnRH neurons is higher in the breeding season [20]. The lower levels of kisspeptin seen during the non-breeding season can be countered by infusion of kisspeptin, which causes ovulation in seasonally acyclic females [26]. Interestingly, the kisspeptin response (stimulating GnRH) is greater during the non-breeding season and this may be due to higher kisspeptin receptor (Kiss1r) expression on GnRH neurons at this time [27].

We hypothesized that the male effect in anestrous ewes is due to activation of kisspeptin cells. This was tested by measuring LH responses to male introduction with and without infusion of a kisspeptin antagonist (P-271). Furthermore we explored the activation of key brain regions after the male stimulus and specifically determined whether kisspeptin neurons in the ARC become activated in response to the male effect. *Kiss1* mRNA was also assessed. Finally, because virtually all kisspeptin neurons in the ARC coexpress neurokinin B (NKB) [28], and NKB activity in kisspeptin neurons is thought shape the physiological regulation of GnRH pulses [29], we examined the expression of the NKB gene *tachykinin 2* (*tac2* mRNA) on *Kiss1* neurons.

## Materials and Methods

### Ethics Statement

Experiments were carried out according to the National Health and Medical Research Council/Commonwealth Scientific and Industrial Research Organisation/Australian Animal Commission Code of Practice for the Care and Use of Animals for Experimental Purposes and were approved by the Monash University, School of Biomedical Sciences Animal Ethics Committee.

### Animals and Peptides

Corriedale ewes of similar age (5–6 years) and weight were maintained at the Monash University Sheep Facility (Werribee, Vic., Australia) under natural conditions of ambient photoperiod and environmental temperature. The ewes had been isolated from males for 2 months and were studied during the anestrous season at this location (December). Kisspeptin antagonist (P-271) was synthesized by EZBiolab Inc. (Carmel, IN), with the sequence: ac-(DA) NWNGFG(D-W)RF-NH2 [30] and the NH2 terminal addition of a seven-amino acid cationic cell-penetrating peptide penetratin RRMKWKK, through an additional tyrosine residue This peptide is efficacious as a kisspeptin antagonist in rats and sheep [16], [31].

### Experimental Design

#### Experiment 1: kisspeptin signaling is critical for the male effect in anestrus ewes

To examine the role of kisspeptin in transmission of the male effect, we administered the kisspeptin antagonist P-271 or vehicle to anestrous ewes prior to and during exposure to a male. Indwelling lateral ventricle (LV) cannulae were implanted into all ewes as described previously [16]. Approximately 2 wk after LV surgery, the animals were housed in individual pens and one external jugular vein was cannulated for blood sampling. The following day, infusion pumps (MS16A; Graseby Medical Ltd., Gold Coast, Australia) were connected to LV cannulae and blood samples (5 ml) collected every 10 min for 6 h. After 2 h, ewes received LV infusions (200 µl/h) of either P-271 (300 µg/h, with a loading dose of 200 µg; n = 4) or vehicle (artificial cerebrospinal fluid, aCSF; n = 4). After 3 h (1 h after commencement of infusion), females were exposed to a novel male, which was able to freely access the front of each single pen, and blood sampling continued for a further 3 h (the male remaining in place). An additional control group (n = 4), remained completely isolated from a male for the entire sampling period and also received aCSF. Plasma was harvested immediately and frozen at −20 C until assayed.

#### Experiment 2: Neural activation following the male effect

At the completion of Experiment 1, ewes (n = 4 per group) were euthanized by an intravenous overdose of sodium pentobarbital (Lethabarb; Virbarc, Peakhurst, N.S.W., Australia). Heads were perfused and the hypothalami dissected as previously described [21]. Coronal sections (30 µm) were cut on a cryostat and placed into cryoprotectant (30% ethylene glycol, 20% glycerol in sodium phosphate buffer) until used for immunohistochemistry or in the same cryoprotectant with 2% paraformaldehyde until used for *in situ* hybridization (all sections stored at −20 C).

### LH Radioimmunoassay

Plasma LH concentrations were measured in duplicate, using the method of Lee et al. [32]. Assay sensitivity was 0.1 ng/ml and the intra-assay coefficient of variation (CV) was less than 10% over the range of 0.6–14.8 ng/ml.

### Immunocytochemistry

*Single-label Fos immunocytochemistry.* Representative sections of the rostral, middle, and caudal regions of the ARC ∼300 µm apart [21], the paraventricular nucleus (PVN), and 3 sections through the mPOA and the ventromedial hypothalamus (VMH) (∼100 µm apart) were chosen from each ewe. Free-floating sections were washed in 0.1 M phosphate-buffered saline (PBS), and incubated in the following reagents: 3% H_2_O_2_, 0.1% sodium borohydride, blocking serum (PBS, 10% normal goat serum, 0.3% Triton X-100), and then a rabbit polyclonal antibody raised against Fos protein (1:20,000, 3 days at 4 C; sc-52, Santa Cruz Biotechnology, Santa Cruz, CA, USA). Fos was visualized with a goat anti-rabbit secondary antibody (1:400, Vector, Burlingame, CA, USA), streptavidin HRP (Vector) and nickel-enhanced DAB (Vector). Sections were mounted onto gel-coated slides, left to dry overnight and then coverslipped. Fos immunoreactive (ir) cells were counted subjectively by an experienced individual blind to the condition of the animal and the number of cells per ewe in each region was averaged to produce a mean (±SEM).

#### Kisspeptin/Fos double-label immunocytochemistry

Sections representing the rostral, middle, and caudal regions of the ARC (as above) were chosen from each ewe and mounted on SuperFrost slides. Immunocytochemistry was performed as previously described [21]. The Fos labeled cells (as above) were visualized with nickel-enhanced DAB (Vector). A rabbit polyclonal antibody against ovine kisspeptin-10 (no. AC566) was used at a dilution of 1:20,000 for 72 h at 4 C and was a gift from A. Caraty (Université Tours, Nouzilly, France) previously validated for use in sheep tissue [18], [28]. Kisspeptin-ir cells were visualized with DAB and counted by an individual blind to the condition of the animal. The total number of kisspeptin-ir cells was recorded as well as those containing Fos (as above), and a percentage colabeled was calculated for each ewe. The percentage of kisspeptin/Fos-ir cells per ewe in each region was averaged to produce a mean (±SEM).

#### GnRH/Fos double-label immunocytochemistry

Sections through the POA (6 sections ∼100 µm apart) were chosen from each ewe. Immunocytochemistry was performed as above. The primary antibody against Fos was visualized with nickel-enhanced DAB and GnRH neurons were visualized with a rabbit polyclonal antibody against GnRH (LR1; kindly supplied by Dr. Robert Benoit, Montreal General Hospital, Canada) and DAB. The total number of GnRH-ir cells was recorded as well as those containing Fos (as above). Percentage colabeled was calculated for each ewe to produce a mean (±SEM).

### Kiss1 mRNA single-label in situ hybridisation

Single-label *in situ* hybridization using ^35^S *Kiss1* antisense riboprobe was performed as described previously [19] on three representative sections of the rostral, medial and caudal regions of the ARC (as above). *Kiss1* mRNA-containing cells were identified under dark-field illumination, and analysis carried out with software designed to count the total number of cells and the number of silver grains per cell, a semiquantitative index of mRNA expression/cell (Image-Pro Plus). Cells were counted when the silver grain density was greater than three times background. For each animal, the number of *Kiss1* cells was counted. Data are expressed as the mean number of identifiable cells and the mean number of silver grains per cell.

### Kiss1 and Tac2 mRNA double-label in situ hybridization

*In situ* hybridization was performed following a standard protocol using ^35^S labeled and digoxigenin (DIG)-labeled riboprobes [16], [33]. In brief, DIG-*Kiss1* and ^35^S-*Tac2* riboprobes were used. The *Tac2* specific sequence spanned bases 31 to 262 of the partial ovine *preprotachykinin 2* sequence (GenBank accession no. AJ507210) and riboprobes synthesized using RT-PCR. Primers were designed with T7 and SP6 promoter sequences attached at the forward and reverse primers, respectively. Three representative sections of the rostral, medial and caudal regions of the ARC (as above) were used for hybridization. *Kiss1* mRNA-containing cells were identified under bright-field illumination (visualized with nitroblue tetrazolium), and *NKB* mRNA analysis carried out with silver grain counting software (as above, Image-Pro Plus). For each animal, the mean silver grain density was counted. Data are expressed as the mean number of silver grains (*Tac2* mRNA) per kisspeptin cell.

### Data Analysis

In experiment 1, LH pulse analysis was performed based on the method described [16]. The effect of male exposure was determined by comparing mean LH, LH pulse amplitude and pulse frequency in the 3 h time periods before (0–180 min), and during male exposure (180–360 min). For pulse frequency, the total number of pulses for each animal was counted and a mean (±SEM) was determined for each 3 h interval. Data were initially examined by two-way ANOVA (effects before and after male exposure), and then one-way ANOVA, using Tukey's multiple comparison post hoc test, was used to determine the effect of male exposure and/or antagonist treatment. Histological data were assessed by one-way ANOVA. Single-label Fos data were log-transformed to achieve homogeneity of varience. Differences were considered significant at the level of P<0.05.

## Results

### Kisspeptin signaling is critical for the male effect in anestrus ewes

**Figure 1:**
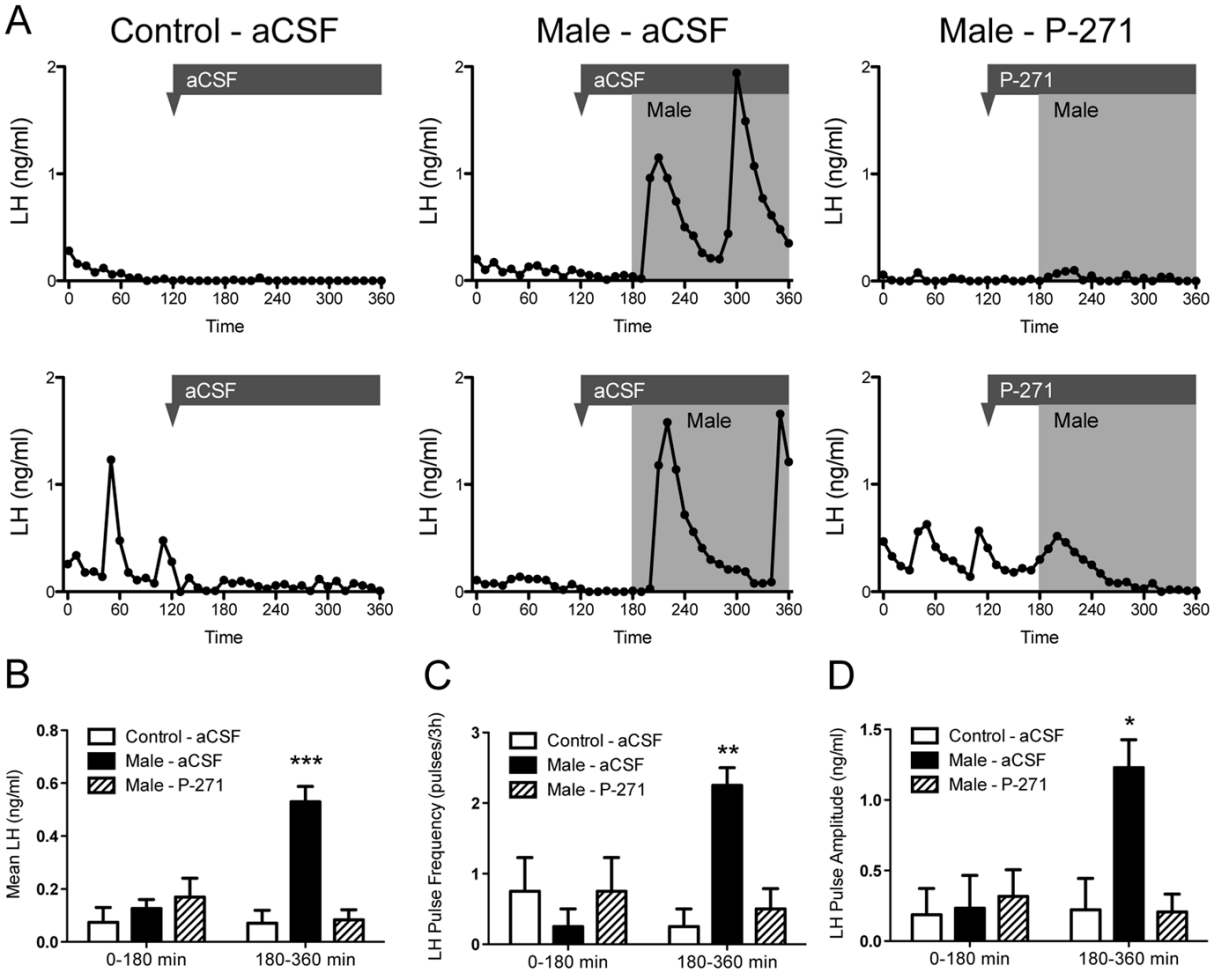
Central infusion of kisspeptin antagonist (P-271) blocks the male effect in anestrous ewes. A, Plasma LH profiles are shown in representative animals treated with artificial cerebrospinal fluid (aCSF) or P-271. The timing of infusion is indicated by the arrowhead/bar, the shaded area indicates male exposure. B–D, Mean LH, LH pulse frequency and pulse amplitude post male exposure. Data are the mean ± SEM, *P<0.05, **P<0.01, ***P<0.001, n = 4 per group.

LH profiles are shown for 6 representative ewes in Figure 1A and were typical for anestrous ewes in the control aCSF group and all animals prior to male exposure. Pulsatile secretory episodes of LH were evident in animals immediately following the introduction of males (mean onset of the first pulse 17.5 ± 4.8 min post-male exposure) and infusion of P-271 abolished this effect (Figure 1A). Mean plasma LH levels (Figure 1B), LH pulse frequency (Figure 1C), and LH pulse amplitude (Figure 1D) increased in response to male exposure and were significantly higher (P<0.05) than in control aCSF animals. Pulse parameters in P-271-infused animals were similar to those in control aCSF-infused animals (Figures 1B–D).

### Neural activation following the male effect

**Figure 2:**
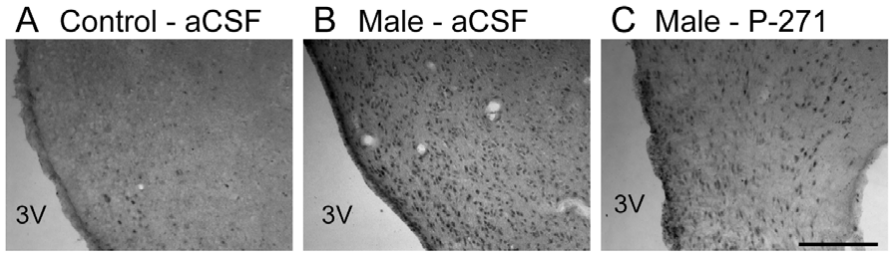
Fos induction in the rostral ARC following male exposure. Representative bright-field photomicrographs showing greater Fos immuno-localization in anestrous ewes exposed to males, with or without kisspeptin antagonist treatment (Male - aCSF, Male - P-271), compared to ewes not exposed to males (Control – aCSF). 3V, Third ventricle. *Scale bar*, 200 µm.

The number of cells displaying Fos in the rostral and mid ARC, was significantly (P<0.05) higher in ewes exposed to the male than control ewes (Figure 2, Table 1). This greater number of Fos positive cells was similar in male-exposed ewes treated with P-271 (Table 1). In the VMH, male exposure also resulted in a greater number of Fos-ir cells, but this was prevented by infusion of P-271 (Table 1). Male exposure had no significant effect on Fos induction in the mPOA, caudal ARC, or the PVN.

**Table 1:** Number of Fos-ir cells in anestrous ewes exposed to males and treated with a kisspeptin antagonist (P-271) or vehicle (aCSF).

	Control - aCSF	Male - aCSF	Male - P-271
**mPOA**	60 ± 19	164 ± 76	120 ± 28
**Rostral ARC**	10 ± 3^a^	103 ± 48^b^	64 ± 19^b^
**Mid ARC**	25 ± 8^a^	134 ± 55^b^	110 ± 47^ab^
**Caudal ARC**	26 ± 7	65 ± 24	155 ± 92
**VMH**	15 ± 6^a^	93 ± 47^b^	45 ± 20^a^
**PVN**	16 ± 3	31 ± 8	24 ± 7

Data are the mean ± SEM (n = 4 per group). Values within each row without common superscripts differ significantly, P<0.05 one-way ANOVA.

### Activation of kisspeptin neurons in the ARC following the male effect

**Figure 3:**
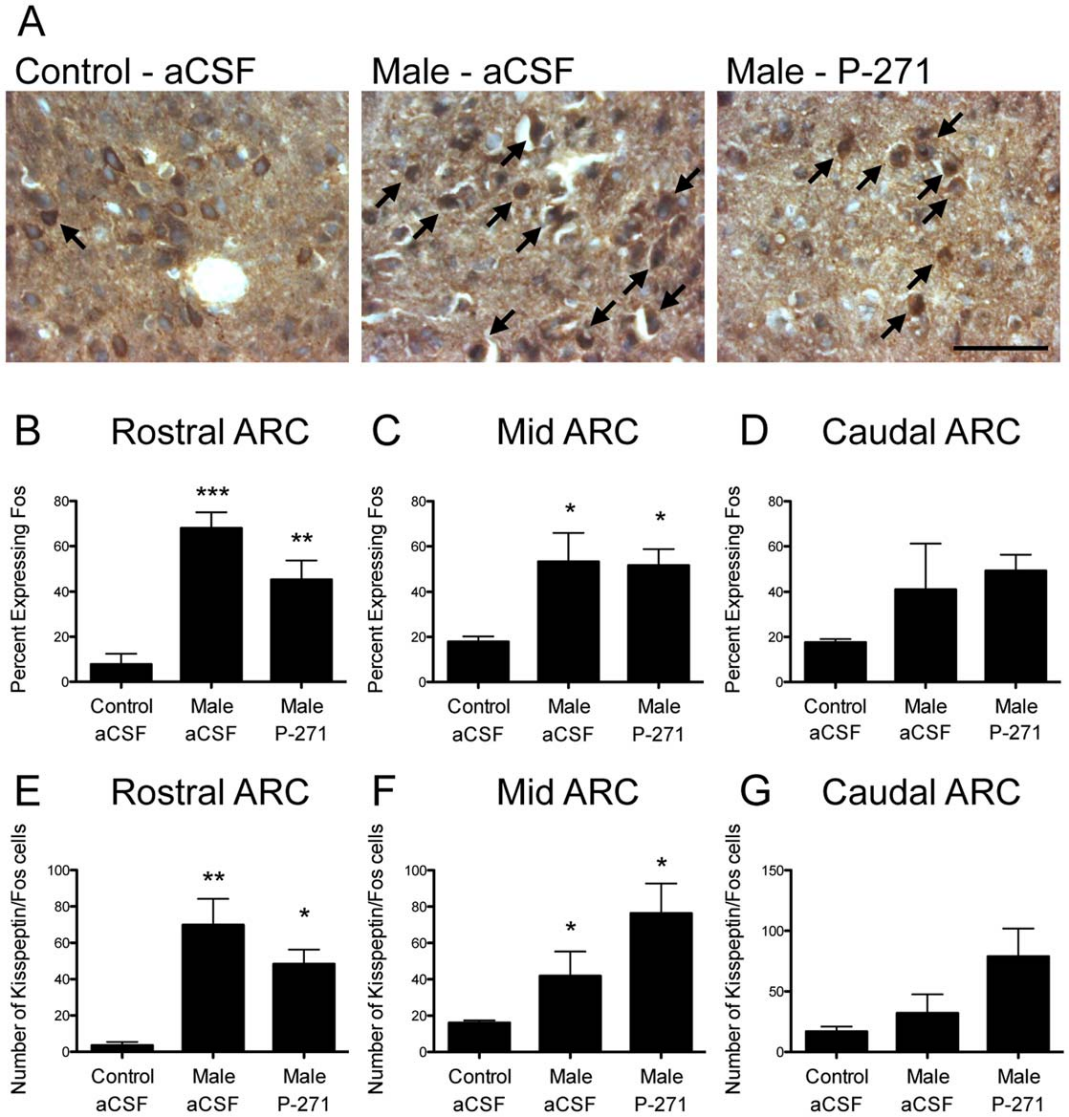
Male exposure in anestrous ewes resulted in Fos induction in ARC kisspeptin neurons. A, Bright-field photomicrographs indicate kisspeptin (brown) and Fos (black) neurons (*arrows*) in the rostral ARC. *Scale bar*, 50 µm. B–D, In the rostral (B) and mid (C) ARC, the percentage of kisspeptin cells co-expressing Fos was greater in male exposed ewes (note: kisspeptin antagonist treatment did not alter Fos induction in kisspeptin cells). No change was evident in the caudal ARC (D). E-G, The number of kisspeptin/Fos neurons was also higher in male exposed ewes in the rostral (E) and mid (F) ARC. Data are the mean ± SEM, *P<0.05, n = 4 per group.

Kisspeptin cells co-expressing Fos were readily detected in the ARC of ewes (Figure 3A). In the rostral ARC, male exposure in anestrous ewes resulted in a 9-fold increase in the percentage of kisspeptin cells co-labeling for Fos compared with controls (P<0.001, Figure 3B). Infusion of P-271 did not prevent the increase in kisspeptin neuron activation following male exposure. Similar results were seen in the mid ARC (3-fold increase in male-exposed ewes, P<0.05, Figure 3C) and there was no change in the caudal ARC (Figure 3D). The number of kisspeptin/Fos neurons also increased following male exposure in the rostral (P<0.01, Figure 3E) and mid (P<0.05, Figure 3F) ARC and infusion of P-271 did not prevent this increase. No change was again noted in the caudal ARC (Figure 3G).

### Activation of GnRH neurons in the mPOA following the male effect

**Figure 4:**
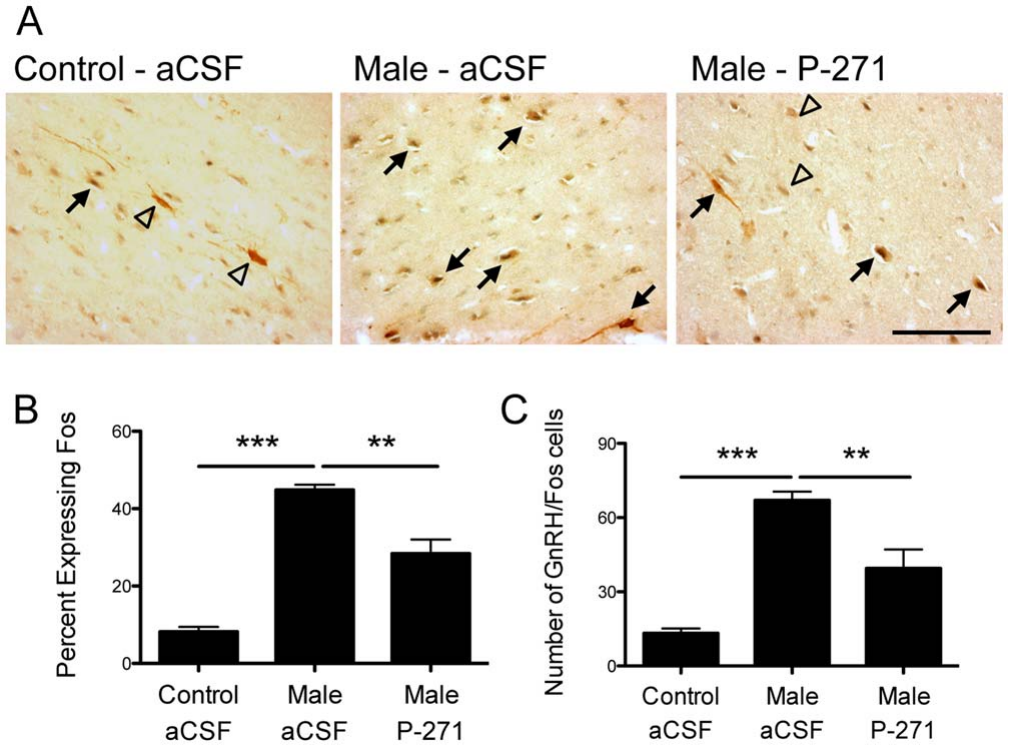
Male exposure in anestrous ewes resulted in Fos induction in GnRH neurons. A, Bright-field photomicrographs indicate GnRH (brown) and Fos (black) neurons (*arrows*) in the mPOA. Single labled GnRH neurons are indicated by *open triangles*. *Scale bar*, 100 µm. B–C, The percentage of GnRH neurons co-expressing Fos and the number of GnRH/Fos neurons was greater in male exposed ewes compared to ewes not exposed to males (Control – aCSF). Kisspeptin antagonist treatment (P-271) reduced the percentage and number of GnRH cells co-expressing Fos in male exposed ewes. Data are the mean ± SEM, ***P<0.001, **P<0.01, n = 4 per group.

Although there was no overall change in Fos labeling in the mPOA, there was a trend for an increase following male exposure. To test whether a sub-population of these Fos-ir cells were GnRH neurons, we examined the co-expression of GnRH and Fos in the mPOA (Figure 4). Male exposure in anestrous ewes resulted in a 5-fold increase in the percentage of mPOA GnRH cells co-labeling for Fos compared with controls (P<0.001, Figure 4). Infusion of P-271 did not prevent GnRH neuron activation following male exposure, but the percentage was significantly less (P<0.01) than that seen in male exposed, aCSF treated ewes. The number of GnRH/Fos neurons mirrored that of percentage expressing FOS (Figure 4 C).

### Kisspeptin expression in the ARC following the male effect

**Figure 5:**
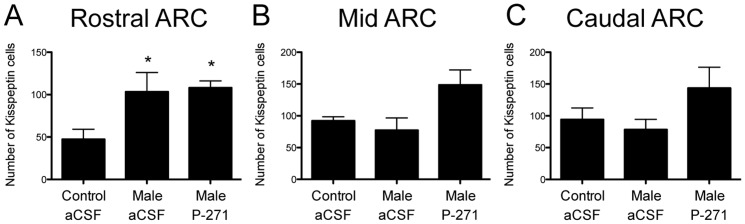
Male exposure in anestrous ewes increased the number of kisspeptin cells in the rostral ARC. The number of detectable kisspeptin neurons in the rostral ARC (A) was higher (P<0.05) in ewes exposed to males compared to control aCSF treated ewes. The number of kisspeptin neurons did not differ in the mid (B), or caudal (C) ARC. Data are the mean ± SEM, n = 4 per group.

*Kisspeptin protein expression.* Kisspeptin-ir neurons in the ARC were examined following kisspeptin/Fos double-label immunocytochemistry. The number of kisspeptin neurons in the rostral ARC was higher (P<0.05) in ewes exposed to males compared to control aCSF treated ewes (Figure 5A). The number of kisspeptin neurons in the mid and caudal ARC did not differ with male exposure or treatment with the kisspeptin antagonist P-271 (Figure 5B, C). To confirm the change in kisspeptin protein expression, we repeated the experiment using single-label kisspeptin immunocytochemistry (Supporting Figure S1).

#### Kiss1 mRNA expression

**Figure 6:**
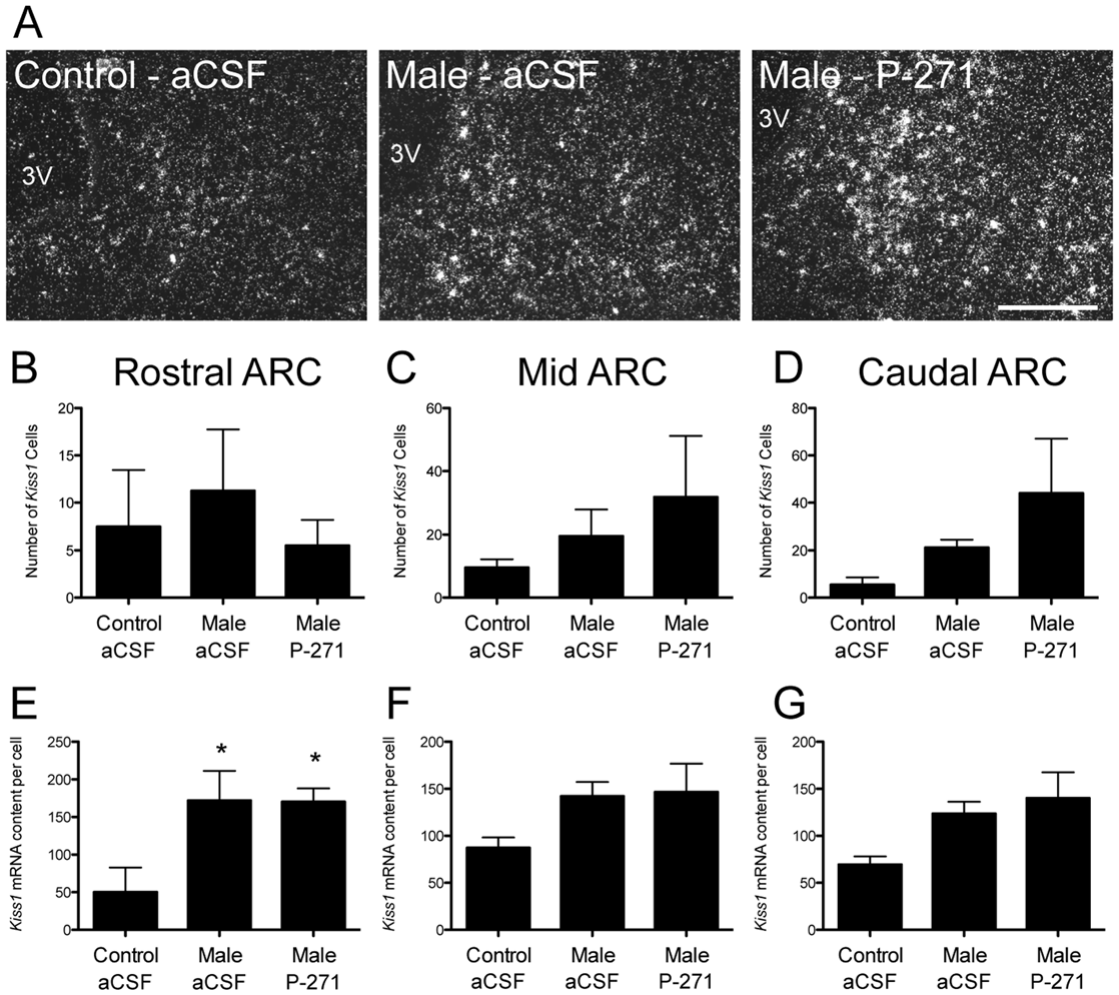
Male exposure in ewes increased the content of *Kiss1* mRNA in the rostral ARC. A, Representative dark-field photomicrographs of the rostral ARC showing *Kiss1* mRNA expressing neurons (as indicated by the presence of silver grain clusters). 3V, Third ventricle. *Scale bar*, 200 µm. B–C, *Kiss1* mRNA content per cell was significantly greater in anestrous ewes exposed to males compared to non-exposed controls in the rostral ARC (B). P-271 had no effect on *Kiss1* mRNA content. Data are the mean ± SEM, *P<0.05, n = 4 per group.

Cells expressing *Kiss1* mRNA were identifiable in the ARC (Figure 6A). The number of *Kiss1* expressing cells was similar in all groups (Figure 6B–D). In the rostral ARC, the *Kiss1* mRNA content per cell was 3-fold greater (P<0.05, Figure 6E) in ewes exposed to a male compared to control ewes. P-271 treatment did not alter this response to male exposure. Similar trends in the mid and caudal ARC were not statistically significant (Figure 6F, G).

### Tac2 mRNA expression on kisspeptin neurons following the male effect

**Figure 7:**
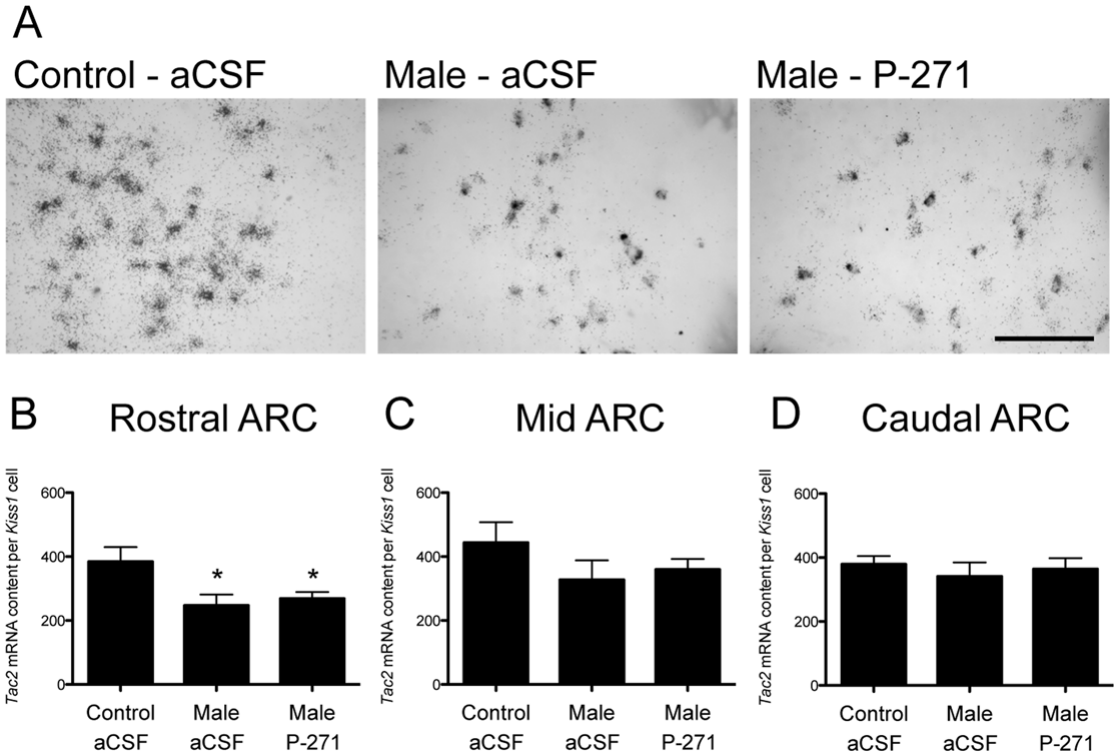
Male exposure in ewes decreased the content of *Tac2* mRNA in the rostral ARC. A, Representative bright-field photomicrographs showing neurons in the rostral ARC containing *Kiss1* mRNA (grey) and *Tac2* mRNA (as indicated by the presence of silver grain clusters). 3V, Third ventricle. *Scale bar*, 50 µm. B–C, *Tac2* mRNA content per *Kiss1* cell was significantly reduced in anestrous ewes exposed to males compared to non-exposed controls in the rostral ARC (B). P-271 had no effect on *Tac2* mRNA content. Data are the mean ± SEM, *P<0.05, n = 4 per group.

*Tac2* mRNA expression was readily detectable in virtually all *Kiss1* mRNA neurons in the ARC (Figure 7A). The per *Kiss1* cell content of *Tac2* mRNA was 36% lower in ewes exposed to males compared to controls (P<0.05, Figure 7B). Kisspeptin antagonist (P-271) treatment did not alter this male exposure effect. The per *Kiss1* cell content of *Tac2* mRNA in the mid and caudal ARC did not differ with male exposure or treatment with P-271.

## Discussion

The LH response in anestrous ewes to the introduction of a male is clearly dependant upon kisspeptin signaling. In addition, we have confirmed the neuronal induction of key hypothalamic nuclei (by measuring Fos-ir) and show the specific activation of kisspeptin neurons in the ARC following the introduction of females to a male. Murata and colleagues [8] first implicated kisspeptin neurons in the promulgation of the pheromone effect in goats by recording MUA in the ARC. Although these data could be considered circumstantial, our present data provide unequivocal proof that the ‘male effect’ depends upon kisspeptin signaling. We have shown this in two ways, 1) prevention of the response *in vivo* with a kisspeptin antagonist and 2) activation of kisspeptin neurons following male introduction demonstrated by Fos activation in these cells.

The effect of a male on anestrous ewes was first described in 1954 [3], and it has since become apparent that the introduction of a ram can override the normal suppressive (negative feedback) effects of estradiol on pulsatile LH secretion in ewes during the non-breeding season [34]. Termed the “male effect”, this sociosexual stimulus has been subsequently examined and refined to show that the key stimulus is an olfactory ‘pheromone’ signal [35]. However, other signals such as visual cues [36] and social stimuli [7]; and finer details, such as male novelty [37] and sexual experience [38] are known that play a significant (albeit lesser) role in the LH response. The effect of pheromones on mammalian reproduction is well recognized [39], [40], but the precise neuroanatomical pathways to the relevant hypothalamic centers and/or GnRH neurons are not defined. Moreover, there is still debate as to whether the male effect in ewes fits the classical definition of a pheromone response [41]. Despite this, the effect of male sociosexual stimuli on LH pulses in anestrous ewes is clear and we now show the vital role of kisspeptin neurons in the ARC of the hypothalamus in this response.

A defining characteristic of the male effect is its rapid onset [34]. In our study, LH pulses were detected within the first 20 min following male introduction, with similar findings reported previously [36], [37], [42]. Such an effect may be indicative of a direct effect of kisspeptin neurons on GnRH soma and/or terminals located in the median eminence. Regarding the latter, direct kisspeptin ‘axoaxonic’ regulation of GnRH secretion was first suggested in mice [43] and the possibility extended to the sheep median eminence [16]. Kisspeptin neurons project to the median eminence in sheep, where varicose fibers come into close apposition to GnRH fibers [16]. Importantly, MUA recordings (possibly reflecting the activity of ARC kisspeptin neurons) show an immediate response following male pheromone exposure in female goats [8]. Furthermore, GnRH neurons respond almost immediately to kisspeptin treatment in mouse brain slice preparations [44] and GnRH secretion into the portal blood is detectable immediately after exogenous kisspeptin administration [16]. Thus, it appears pheromones recruit kisspeptin neurons almost immediately and the very rapidly resulting GnRH/LH pulses are likely to be promulgated directly within GnRH neuron soma and/or terminals in the median eminence. Consistent with this scenario, the GnRH response to kisspeptin appears to be primed in anestrous ewes, with greater secretion of GnRH measured in the portal circulation in response to exogenous kisspeptin treatment and higher expression of Kiss1r mRNA in GnRH neurons during the non-breeding season [27].

The neuronal pathways involved in the transmission of pheromone signals through the olfactory lobes on route to the reproductive centers of the brain (via the main and accessory olfactory systems) have been extensively described and reviewed [4], [5], [6]. The pathway signals take within the hypothalamus, prior to GnRH neuron activation, requires further investigation. Previous studies have utilized histological assessment of neural activation, via the visualization of Fos protein, and the male effect in ewes resulted in activation of cells in the POA and VMH [7]. Another study yielded similar results in the POA and VMH, but noted that the number of activated cells increased over time, such that a greater number of Fos-ir cells were observed 6 h after male exposure compared to 2 h [41]. Our data show Fos activation of neurons in the rostral and mid ARC after 3 h of male exposure, which has not previously been shown following the male effect (although some studies did not appear to examine the ARC [7]). This inconsistency could relate to the differing timeframes of male exposure. Moreover, our data indicate that a number of these activated ARC cells are kisspeptin neurons. However, it should be noted that when the absolute number of activated kisspeptin neurons is compared to the total number of Fos positive cells in the ARC, it is clear that kisspeptin neurons are not the sole neuronal population in the ARC involved in the male effect. As in earlier studies, we saw significant activation of neurons in the VMH associated with the male effect. Moreover, kisspeptin antagonist treatment appeared to prevent the effect. Thus, kisspeptin signaling could directly regulate VMH activation following the male effect. This appears unlikely, because there is no documented expression of Kiss1r in the VMH, at least in the mouse [45]. Alternatively, VMH activation may be due to a stimulus downstream from kisspeptin signaling or even due to the predicted rise in estradiol–the VMH is rich in cells expressing estrogen receptor [46]–in ewes exposed to males.

Increased *Kiss1* mRNA cellular content was evident in the rostral ARC following the male effect and was consistent with the increase in transcriptional activation of kisspeptin cells in this region. Conversely, we saw a decline in the content of *Tac2* mRNA in kisspeptin neurons in the rostral ARC following male exposure. These data were unexpected because NKB (the product of the *Tac2* gene) is thought to stimulate kisspeptin neurons [47] and shape the physiological regulation of GnRH pulses [29]. Indeed, a recent study shows NKB administration to anestrous ewes activates kisspeptin neurons and stimulates LH secretion [48]. Virtually all kisspeptin neurons in the ARC coexpress NKB and dynorphin [28], leading to the KNDy (Kisspeptin, NKB, Dynorphin) neuron terminology [29]. In spite of this, the ability of NKB to stimulate LH secretion in sheep was recently shown to arise from the retrochiasmatic area of the hypothalamus [49] not the ARC. Alternatively, data in rats suggest NKB inhibits LH pulses and multiunit activity volleys [50], [51] and in humans the concept of KNDy neurons has recently been challenged [52]. Upon closer inspection, it appears the inhibitory effects of NKB on LH secretion may be dependant on the prevailing sex-steroid milieu, where it has been shown that low or absent estrogen levels facilitate negative effects [53]. Given this, and our data, it is possible that in the anestrous ewe (with low levels of estradiol) NKB may have predominantly negative effects. Clearly, these data illustrate the complexity and diversity of the NKB system and further investigation of the actions of NKB in sheep is warranted.

In our study, kisspeptin neuron activation and *Kiss1* mRNA content per cell responses to the male effect were restricted the rostral to mid regions of the ARC (not the caudal area) and were unaffected by kisspeptin antagonist treatment. In regard to the former, this is consistent with the proposed role for the ARC in estradiol negative feedback. Our previous data show that *Kiss1* mRNA expression in the caudal region of the ARC is important for the generation of the estrogen positive feedback preovulatory GnRH/LH surge in the ewe [17], [21] and *Kiss1* in the rostral to mid regions of the ARC appears to be more responsive to negative feedback [20], [21], although *Kiss1* mRNA content appears to be up-regulated in the rostral ARC at the time of estrous [17]. It is conceivable that the kisspeptin neurons in the rostral to mid ARC are predominantly recruited in estrogen negative feedback control and the tonic control of GnRH/LH pulses; our data are consistent with this proposition as these neurons are activated following the male effect, thus releasing seasonal negative feedback and allowing GnRH secretion. In regard to the latter, kisspeptin antagonist treatment did not prevent the changes in rostral to mid ARC kisspeptin neurons following the male effect. This was expected because the competitive antagonist prevents kisspeptin signaling at the receptor level by binding to—and not activating—Kiss1r, thus any changes in kisspeptin neurons *per se* would be unimpeded.

In our experiments, we focused on the effects of male exposure on kisspeptin neurons in the ARC. In sheep, a significant population of kisspeptin neurons is also located in the dorso-lateral POA [18], [19], [20]. However, these neurons only appear to be involved in estrogen positive feedback and the generation of the GnRH/LH surge [21], [54]. Indeed, manipulations in negative feedback (ovariectomy and chronic estradiol replacement) during the breeding season did not alter Fos induction in POA kisspeptin neurons, but did so in ARC kisspeptin neurons, consistent with the increase in pulsatile GnRH/LH secretion [21]. Despite this we did see a trend for an increase in Fos induction within unidentified cells of the mPOA in anestous ewe exposed to males. We believe this to be representative of GnRH neurons, and we further show an increase in GnRH neuron activation following male exposure, which was reduced (but not abolished) by kisspeptin antagonist treatment. The latter is intriguing, given that kisspeptin antagonist treatment completely abrogated LH pulses. It is possible that the level of GnRH activation following kisspeptin antagonist treatment is unable to support the production of GnRH/LH pulses or it may indicate that GnRH neuron activation following the male effect is mediated by other factors, in addition to kisspeptin signaling, which do not directly result in GnRH/LH release. Alternatively, neuronal activation in the POA following the male effect may indicate the onset of a positive feedback “surge-like” response. Although the male effect is known to induce GnRH/LH surges in females [55], we feel this is unlikely to be a direct effect of male exposure and more likely due to the subsequent release of negative feedback. The activation of rostral to mid ARC kisspeptin neurons is consistent with this and a similar phenomenon has been reported with exogenous kisspeptin treatment in anestrous ewes [56].

In this experiment, we focused on the GnRH neurons within the mPOA. This mPOA population represents the majority of GnRH neurons within the ovine brain and project to the median eminence [57], [58]. Alternatively, it is suggested that separate populations of GnRH neurons are associated with the pulsatile and surge release of GnRH; and it is GnRH neurons located in the mediobasal hypothalamus that are the conduit for pulsatile secretion. This evidence comes from studies where increased LH pulses, induce by opioid antagonist treatment in females, induced Fos expression in mediobasal hypothalamic GnRH neurons, but not GnRH neurons in the POA [59]. Our data do not agree, and are similar to data from Gelez and Fabre-Nys [7] who show an increase in LH pulses after male exposure in anestrous ewes paired with activation of GnRH neurons in the POA. However, it remains possible that the small population of GnRH neurons within the mediobasal hypothalamus become activated by male exposure.

Overall, this study is the first to use an ovine model to investigate the vital role of kisspeptin signaling in mediating the effect of pheromones, via the male effect, and in-turn identify kisspeptin neurons in the brain as key targets for pheromone activation. The results allow us to further understand the pathways in the brain, and particularly the hypothalamus, which are activated by pheromones to stimulate the reproductive system. We conclude that kisspeptin signaling is vital in the transmission of the stimulus from male sheep that activates reproductive centers in the brain of anestrous females to elicit pulsatile GnRH secretion.

## Supporting Information

Figure S1 **Male exposure in anestrous ewes increased the number of kisspeptin cells in the rostral ARC.** Sections representing the rostral, middle, and caudal regions of the ARC (as above) were chosen from each ewe and mounted on SuperFrost slides. Fluorescent immunocytochemistry was performed as previously described [20]. The primary kisspeptin antibody (AC566) was used at a concentration of 1:2000 and was visualized with a goat anti-rabbit secondary antibody (Alexa 448, 1:400; Molecular Probes Inc., Eugene, OR). Kisspeptin-ir cells were identified under fluorescent illumination, with a single observer counting the total number of cells. For each ewe, the number of kisspeptin-ir cells per section in each region was averaged to produce a mean (±SEM). A, Representative photomicrographs of the rostral ARC showing kisspeptin immunoreactive neurons (green). 3V, Third ventricle. *Scale bar*, 200 µm. B-C, The number of detectable kisspeptin neurons in the rostral ARC (B) was higher (P<0.05) in ewes exposed to males compared to control aCSF treated ewes. The number of kisspeptin neurons did not differ in the Mid (C), or Caudal (D) ARC. Data are the mean ± SEM, n = 4 per group.
(TIF)
