# Supplementary figures and images for: Kisspeptin Signaling Is Required for the Luteinizing Hormone Response in Anestrous Ewes following the Introduction of Males
Source: PLoS One. 2013 Feb 28;8(2):e57972. doi: 10.1371/journal.pone.0057972 (PMC3585258; doi:10.1371/journal.pone.0057972)

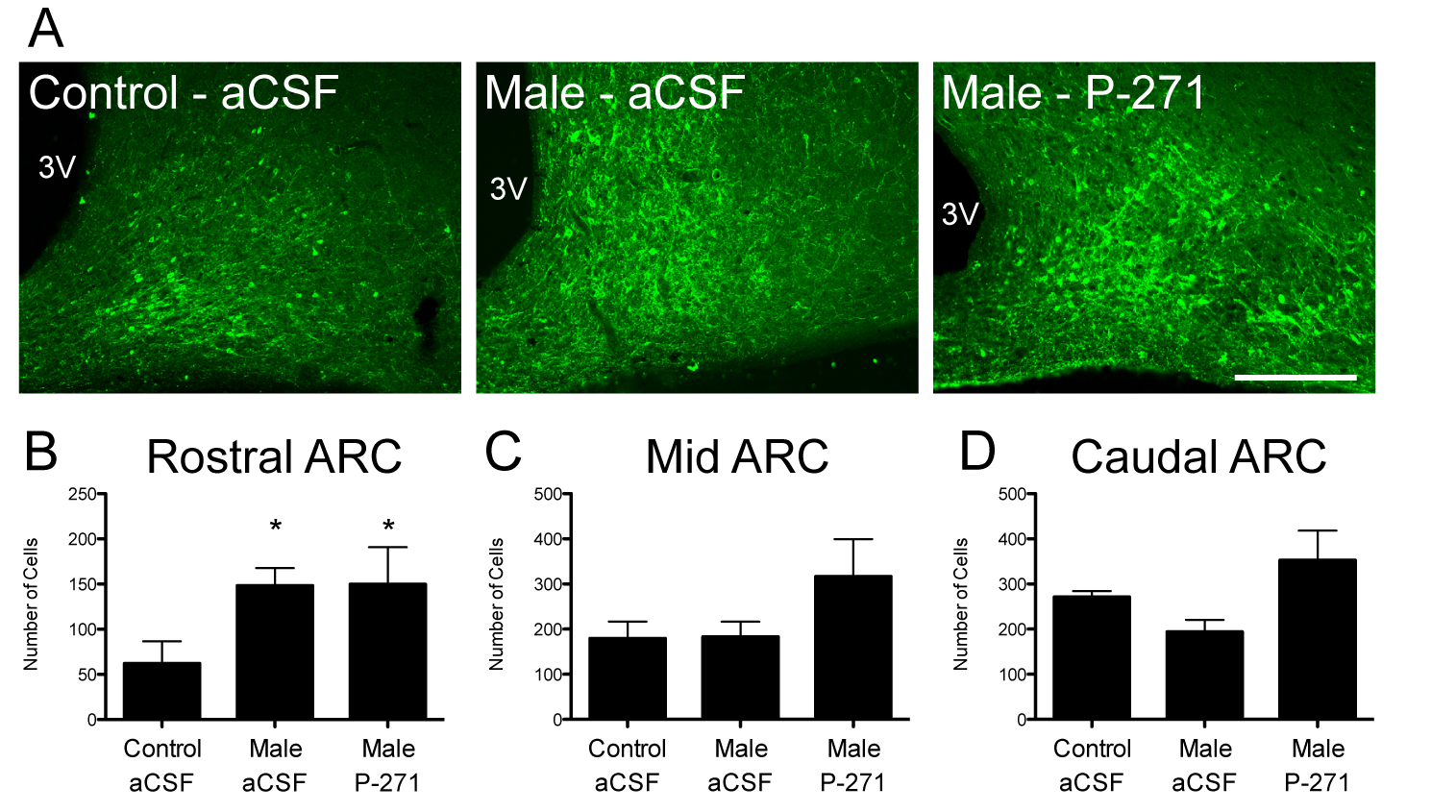

Supplement: Figure S1 — Male exposure in anestrous ewes increased the number of kisspeptin cells in the rostral ARC. Sections representing the rostral, middle, and caudal regions of the ARC (as above) were chosen from each ewe and mounted on SuperFrost slides. Fluorescent immunocytochemistry was performed as previously described [20]. The primary kisspeptin antibody (AC566) was used at a concentration of 1:2000 and was visualized with a goat anti-rabbit secondary antibody (Alexa 448, 1:400; Molecular Probes Inc., Eugene, OR). Kisspeptin-ir cells were identified under fluorescent illumination, with a single observer counting the total number of cells. For each ewe, the number of kisspeptin-ir cells per section in each region was averaged to produce a mean (±SEM). A, Representative photomicrographs of the rostral ARC showing kisspeptin immunoreactive neurons (green). 3V, Third ventricle. Scale bar, 200 µm. B-C, The number of detectable kisspeptin neurons in the rostral ARC (B) was higher (P<0.05) in ewes exposed to males compared to control aCSF treated ewes. The number of kisspeptin neurons did not differ in the Mid (C), or Caudal (D) ARC. Data are the mean ± SEM, n = 4 per group. (TIF) [file pone.0057972.s001.tif]
